# Draft Genome Sequences of *Sphingomonadaceae* Strains Isolated from a Freshwater Lake

**DOI:** 10.1128/mra.00070-22

**Published:** 2022-04-06

**Authors:** Shang Shen, Tetsunobu Anazawa, Tomonari Matsuda, Yoshihisa Shimizu

**Affiliations:** a Research Center for Environmental Quality Management, Kyoto University, Otsu, Shiga, Japan; b Lake Biwa Branch Office, National Institute for Environmental Studies, Otsu, Shiga, Japan; University of Maryland School of Medicine

## Abstract

We isolated two bacterial strains (*Sphingomonadaceae* family) from Lake Biwa, Japan. Based on whole-genome sequencing results, one strain (BSN-002) was assigned to the *Sphingopyxis* genus and the other (BSN-004) to Sphingomonas aquatilis.

## ANNOUNCEMENT

Members of the family *Sphingomonadaceae* (of the order *Sphingomonadales* and the class *Alphaproteobacteria*) are abundant in marine waters, freshwater, and drinking water (1, 2). They degrade refractory organic matter containing monocyclic and polycyclic aromatic hydrocarbons and lignin-derived compounds (3, 4). We isolated two *Sphingomonadaceae* strains from Lake Biwa, Japan.

Our strains (BSN-002 and BSN-004) were isolated offshore (35°23′21.0″N, 136°07′51.0″E) and cultured in modified lysogeny broth (LB) agar medium at 25°C for 1 week. Modified LB agar medium was prepared as LB agar (5) diluted 100-fold. Colonies obtained were restreaked twice on the same medium. An individual bacterial colony was scraped from the agar plate, and DNA was extracted using the DNeasy PowerWater kit (Qiagen, Hilden, Germany) according to the manufacturer’s protocol. The library was prepared using the MGIEasy FS DNA library preparation set, the MGIEasy circularization kit, and the DNBSEQ-G400 high-throughput sequencing set (MGI Tech Co., Shenzhen, China) according to the manufacturer's instructions. The sample was sequenced using the DNBSEQ-G400 system (MGI Tech) with 2 × 200-bp reads. Default parameters were used to construct the bacterial genome except where otherwise noted. Raw reads with low-quality regions were removed using Trimmomatic v.0.39 with default settings (6). Totals of 24,568,383 and 25,858,865 paired-end reads were recovered for BSN-002 and BSN-004, respectively, after trimming. The trimmed reads were assembled using SPAdes v.3.13.1 with the –careful option (7). Two strains were classified using GTDB-Tk v.1.3.0 with the –classify_wf option against the Genome Taxonomy Database (GTDB), release 05-RS95. Genome annotation was conducted using the NCBI Prokaryotic Genomic Annotation Pipeline (PGAP) (8) for BSN-002 and the DDBJ Fast Annotation and Submission Tool (DFAST) (9) for BSN-004.

BSN-002 comprised 3,732,098 bp, with a GC content of 65.3% and average coverage of 992×. A total of 3,623 protein coding sequences (CDSs), 3 rRNAs, and 47 tRNAs were identified. The closest strain was *Sphingopyxis sp001468265* (accession number GCF_001468265.1 in the GTDB), with an average nucleotide identity (ANI) value of 87.3%. This value was lower than the cutoff value for species discrimination (95%), indicating that strain BSN-002 was not assigned to an existing species in the GTDB. BSN-004 comprised 1,128,425 bp, with a GC content of 68.0% and average coverage of 933×. A total of 1,067 CDSs and 14 tRNAs were identified. The closest strain, Sphingomonas aquatilis (accession number GCF_000379045.1 in the GTDB), had an ANI value of 96.3%.

### Data availability.

Raw reads from whole-genome sequencing were deposited in the DDBJ (DRA accession numbers DRA013358 and DRA013359). Assembled contigs for the two strains were deposited in GenBank (accession number CP091804) for BSN-002 and in the DDBJ (accession number BQWF01000001) for BSN-004.
